# Does socio-economic status influence the effect of multimorbidity on the frequent use of ambulatory care services in a universal healthcare system? A population-based cohort study

**DOI:** 10.1186/s12913-021-06194-w

**Published:** 2021-03-06

**Authors:** Cynthia MBUYA-BIENGE, Marc SIMARD, Myles GAULIN, Bernard CANDAS, Caroline SIROIS

**Affiliations:** 1 Department of Social and Preventive Medicine, Faculty of Medicine, Laval University, Quebec, QC Canada; 2 Quebec National Institute of Public Health, Quebec, QC Canada; 3 Centre de Recherche Sur les Soins et les Services de Première Ligne de l’Université Laval, Québec, Canada; 4 National Institute of Excellence in Health and Social Services, Quebec, QC Canada; 5 Centre d’excellence sur le vieillissement de Québec, Centre de recherche du CHU de Québec, Quebec, QC Canada

**Keywords:** Multimorbidity, Socioeconomic status, Ambulatory care utilization, Effect modification, Frequent healthcare users

## Abstract

**Background:**

Frequent healthcare users place a significant burden on health systems. Factors such as multimorbidity and low socioeconomic status have been associated with high use of ambulatory care services (emergency rooms, general practitioners and specialist physicians). However, the combined effect of these two factors remains poorly understood. Our goal was to determine whether the risk of being a frequent user of ambulatory care is influenced by an interaction between multimorbidity and socioeconomic status, in an entire population covered by a universal health system.

**Methods:**

Using a linkage of administrative databases, we conducted a population-based cohort study of all adults in Quebec, Canada. Multimorbidity (defined as the number of different diseases) was assessed over a two-year period from April 1st 2012 to March 31st 2014 and socioeconomic status was estimated using a validated material deprivation index. Frequents users for a particular category of ambulatory services had a number of visits among the highest 5% in the total population during the 2014–15 fiscal year. We used ajusted logistic regressions to model the association between frequent use of health services and multimorbidity, depending on socioeconomic status.

**Results:**

Frequent users (5.1% of the population) were responsible for 25.2% of all ambulatory care visits. The lower the socioeconomic status, the higher the burden of chronic diseases, and the more frequent the visits to emergency departments and general practitioners. Socioeconomic status modified the association between multimorbidity and frequent visits to specialist physicians: those with low socioeconomic status visited specialist physicians less often. The difference in adjusted proportions of frequent use between the most deprived and the least deprived individuals varied from 0.1% for those without any chronic disease to 5.1% for those with four or more chronic diseases. No such differences in proportions were observed for frequent visits to an emergency room or frequent visits to a general practitioner.

**Conclusion:**

Even in a universal healthcare system, the gap between socioeconomic groups widens as a function of multimorbidity with regard to visits to the specialist physicians. Further studies are needed to better understand the differential use of specialized care by the most deprived individuals.

**Supplementary Information:**

The online version contains supplementary material available at 10.1186/s12913-021-06194-w.

## Background

The increasing burden of chronic diseases is a major concern worldwide [1]. A growing number of individuals live with multimorbidity, defined as the presence of two or more long term medical conditions [2, 3]. Multimorbidity challenges healthcare systems which are mostly oriented towards single disease management [4]. Multimorbidity is often managed with an inappropriate use of healthcare services, reflected in fragmented and less efficient care leading to higher costs for health care systems [5]. In fact, multimorbid individuals make a greater use of primary care services such as general practitioners (GP) and emergency departments (ED). They also visit specialized physicians (SP) more often and have more frequent unplanned hospital admissions [3, 6].

Multimorbidity is more prevalent among individuals with a lower socioeconomic status (SES) [7, 8]. Multimorbidity tends to occur approximately 10 years earlier in individuals living in more deprived areas [4]. These individuals present a higher combination of physical and mental illnesses thus increasing the complexity of care [4, 9]. Multimorbid patients with low SES are also more likely to report a lower perceived health and a lower quality of life [10].

Even in universal health care systems, the impact of SES differs according to the category of health services sought by patients. Lower SES has been associated with a higher use of primary care and hospital services while a higher SES has been associated with more specialized care [11, 12]. Addressing socioeconomic disparities in healthcare use is critical to achieve better health outcomes for patients [13]. However, SES is still a major social determinant of health related to inequalities in health which are a main predictor of an individual’s health status throughout their life [14].

With complex healthcare needs, multimorbid individuals with low SES are more likely to make frequent use of primary care services [15]. Frequent healthcare users, often defined as the top 1, 5 or 10% of total users, challenge the sustainability and cost-efficiency of health care systems [16]. From the healthcare system perspective, interventions targeting this small group of the population responsible for a greater use of care have been proposed by other studies in order to help to reduce costs and to better manage resources for the entire population [17, 18].

Several studies have shown the independent association of multimorbidity and SES with ambulatory care use [6, 11, 19–21]. However, the manner those two factors interact to modulate the risk of being a frequent user of healthcare services is still unclear. This population-based cohort study therefore aims to determine the effect modification of SES on the association between multimorbidity and frequent ambulatory care utilization of emergency department, general practitioners and specialist physicians in the adult population.

## Methods

### Data sources

Administrative data were obtained from the Quebec Integrated Chronic Disease Surveillance System (QICDSS) which consists of five health administrative databases linked together using a unique identifier for each individual to monitor chronic diseases and their determinants in the entire population [22]. The QICDSS includes the health insurance registry, the hospital discharge database, the death registry, the pharmaceutical services database and the fee-for-service physician claims database. Because the province of Quebec (Canada) has a universal access to physician services and hospitalizations, the administrative database contained health information for about 7.8 million residents in the 2011–12 fiscal year [22]. Diagnoses in physician claims database are coded using a derived classification of the ninth revision of the International Classification of Diseases (ICD-9). As of April 1st 2006, primary and up to 25 secondary diagnoses in the hospital discharge database are coded using the ICD-10 Canadian coding standards.

### Study design and population

**Fig. 1:**
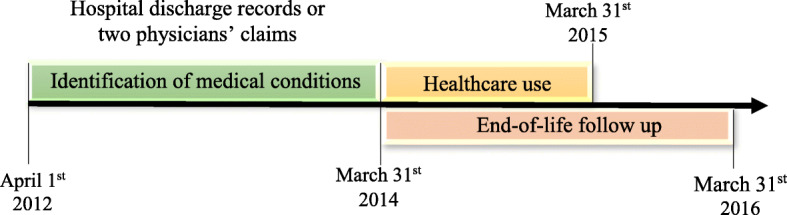
Description of the study period

We performed a retrospective cohort study using the QICDSS from April 1st 2012 to March 31st 2016 of all individuals aged 18 years and older as of October 1st 2014. A flow chart illustrating exclusion of participants is available in additional file 1. To be included, the individuals needed a valid health card for the duration of the study. The cohort includes 94.4% of all Quebecers adult population and is representative of the province population [22]. We excluded all individuals living in long-term health care facilities since their access to care is managed differently and those who died during and 1 year after the follow-up period (between April 1st 2015 and March 31st 2016). The last criteria was used to exclude individuals who might have needed end-of life care, who are unrepresentative of the general population [23] (Fig. 1).

### Variable of interest: multimorbidity

The presence of multimorbidity was evaluated according to the number of diseases presented by each individual. We categorised the number of conditions into five groups (0, 1, 2, 3 and ≥ 4) based upon the distribution of medical conditions in our population in order to have enough statistical power to perform our analyses. We used a disease count from the list of 31 medical conditions of the Combined Comorbidity Index previously validated with the QICDSS [24]. We searched for medical conditions diagnosed between April 1st 2012 and March 31st 2014 (Fig. 1). The presence of at least one diagnosis in a hospital discharge records file or of two physicians’ claims with the same diagnosis within a year and spaced at least 30 days apart were necessary to include the disease into the count. These algorithms increase the validity of the disease identification [25]. We excluded gestational diabetes and hypertension using the presence of a pregnancy related diagnosis during the period.

### Effect modification variable: socioeconomic status

Socioeconomic status (SES) was determined using a validated area-based material deprivation index [26]. This ecological measure assigns to each individual a deprivation quintile (1 = least deprived, 5 = most deprived) using information from income, education and employment indicators of all individuals aged 15 and older living in the same neighbourhood. For example, an individual will be assigned a deprivation quintile of 5 if, in their neighbourhood, the proportion of individuals without a high school certificate or diploma, the proportion of employed people and the average income are below the national median income. Therefore, the material deprivation index provides a territorial measure of the SES.

### Outcomes: frequent use of ambulatory care services

Ambulatory care use was assessed with the number of ED visits, GP visits or SP visits during the follow-up period from April 1st 2014 to March 31st 2015 (Fig. 1). In addition, we calculated the total number of visits to ambulatory care as the sum of the annual visits to these three services. An ED visit was defined by at least one physician claim issued from an ED on up to 2 consecutive days per a validated algorithm [27]. GP or SP visits were obtained from the physician claims as we retained only outpatient visits. Two claims from the same physician on the same day were counted as a single visit. Claims on ED, GP and SP utilization were retrieved from the hospital discharge and the fee-for-service physician claims databases (codes used to identify the healthcare provider and the type of institution are presented in additional file 2). In absence of a consensual definition of frequent users of ambulatory care services, we based our definition using percentile cut-off as done in other studies [11, 17, 28]. The number of annual visits of individuals at the 95th percentile of the population was used to define frequent users within each category of ambulatory services and for the total. Thus, the cut-off number of annual visits of frequent users was ≥3 for ED visits, ≥7 visits for GP, ≥10 visits for SP and ≥ 17 total visits to any ambulatory care services.

### Covariates (adjustment variables)

Covariates included age, sex, social deprivation index and rurality. Social deprivation index is an ecological proxy based on the postal code like the material deprivation index, and is based on the proportion of people living alone at home, separated, divorced or widowed or single-parent families [26]. Rurality was defined as rural communities with less than 10,000 residents. The covariates were selected upon their availability in our databases and their known relation with multimorbidity and frequent healthcare use.

### Statistical analysis

Descriptive statistics were used to characterize the study population and the ambulatory care utilization. Four multivariate logistic regression models, one for each category of ambulatory service and one for the total use, were performed to evaluate the potential effect modification of the SES on the association between multimorbidity and the frequent ambulatory care use. Each model was adjusted for the covariates. We excluded individuals with missing values for the SES. Multicollinearity was checked beforehand between all variables using the Pearson correlation coefficients (no correlation coefficient was over 0.7). We estimated adjusted proportions of frequent users with a logistic regression model using the prediction at the means method [29] and the addition of an interaction term (multimorbidity*SES) to every statistical model. Thus, we modeled the following equation for each ambulatory care services: Frequent use of services (yes/no) = multimorbidity (number of diseases in category) + SES (quintiles of deprivation) + multimorbidity*SES + age + sex + social deprivation + rurality. The effect modification was calculated on an additive scale by comparing graphically if the differences in adjusted proportions of frequent visits were similar for each level of deprivation and if the difference between the most deprived and the least deprived group was similar across the disease count group. Effect modification on the additive scale is relevant in a public health perspective since it estimates, at a population level, the absolute effect of a risk factor on an outcome [30]. As we used a large population database, “statistical significance” was not needed to evaluate the likelihood of the effect modification [31]. Based on graphical representation of the adjusted proportion of frequent users by disease count for each value of the SES, the absence of variation in the distance between the SES lines was indicative of an absence of effect modification on the additive scale. In contrast, non-parallel lines indicated an effect modification. Analyses were performed using SAS 9.4 (SAS Institute, Cary, NC).

### Sensitivity analysis

We performed two sensitivity analyses. First, we replicated the main analysis using the 80th percentile instead of the 95th as a cut-off for frequent healthcare users [28]. Second, we measured multimorbidity using the combined comorbidity score index, a weighted count of the 31 medical conditions validated with the QICDSS [24]. A high comorbidity score indicates a strong association with mortality. We divided the score into five groups (0, 1, 2–3, 4–5, 6+) and proceeded to the analyses previously described.

## Results

Our study population included 5,316,830 individuals with a mean age of 50.7 years (SD 17.9) and 52.2% of female (Table 1). Close to 10% had 2 or more chronic diseases, which is considered multimorbidity. Frequent healthcare users were mostly females, with proportions varying from 56.2 to 67.3%, while the proportions of those with multimorbidity ranged from 21.0 to 35.9%. Frequent users accounted for 25.2% of the 27,712,121 total ambulatory care visits of any category (Table 2). Frequent users of ED, GP and SP were responsible for 41.3, 28.9 and 39.6% of the total annual visits to each service, respectively. Furthermore, frequent users of one category of service were not necessarily the same for the others: less than a quarter of frequent users of one category of service were also frequent users for another category. Yet, most frequent users of total ambulatory care were frequent users of SP (72.0%).

**Table 1:** Characteristics of the total adult population and of frequent users of ambulatory care services in Quebec, Canada for 2014–2015

Characteristics	Total population n, (%)	Frequent users^a^			
		Emergency departments (3+) n, (%)	General practitioners (7+) n, (%)	Specialist physicians (10+) n, (%)	Total ambulatory care services (17+) n, (%)
**Population size**	5,316,830 (100.0)	213,920 (100.0) ^b^	347,370 (100.0) ^b^	307,696 (100.0) ^b^	271,358 (100.00) ^b^
**Age, yr, Mean ± SD**	50.7 ± 17.9	53.9 ± 20.4	54.7 ± 19.2	58.9 ± 17.6	58.0 ± 18.4
**Age groups**					
18–44	1,976,637 (37.2)	74,368 (34.8)	113,083 (32.6)	71,654 (23.3)	70,026 (25.8)
45–64	2,068,153 (38.9)	66,776 (31.2)	119,492 (34.4)	98,639 (32.1)	86,423 (31.9)
65–84	1,137,476 (21.4)	58,801 (27.5)	92,913 (26.8)	125,460 (40.8)	101,019 (37.2)
85+	134,564 (2.5)	13,975 (6.5)	21,882 (6.3)	11,943 (3.9)	13,890 (5.1)
**Sex**					
Female	2,776,966 (52.2)	120,318 (56.2)	233,770 (67.3)	184,796 (60.1)	170,729 (62.9)
Male	2,539,864 (47.8)	93,602 (43.8)	113,600 (32.7)	122,900 (39.9)	100,629 (37.1)
**Material deprivation**					
1 (Least deprived)	998,294 (18.8)	25,972 (12.1)	56,067 (16.1)	60,734 (19.7)	47,999 (17.7)
2	1,026,652 (19.3)	33,079 (15.5)	64,090 (18.5)	57,411 (18.7)	48,968 (18.1)
3	1,019,183 (19.2)	38,503 (18.0)	66,696 (19.2)	57,632 (18.7)	50,732 (18.7)
4	1,020,353 (19.2)	45,851 (21.4)	69,035 (19.9)	57,831 (18.8)	52,645 (19.4)
5 (Most deprived)	968,560 (18.2)	54,363 (25.4)	66,835 (19.2)	55,618 (18.1)	52,412 (19.3)
Missing	283,788 (5.3)	16,152 (7.6)	24,647 (7.1)	18,470 (6.0)	18,602 (6.9)
**Number of medical conditions**					
0	3,810,758 (71.7)	109,021 (51.0)	177,934 (50.2)	113,712 (37.0)	100,573 (37.1)
1	1,013,676 (19.1)	47,962 (22.4)	96,444 (27.8)	85,208 (27.7)	73,216 (27.0)
2	254,778 (4.8)	19,698 (9.2)	34,562 (10.0)	45,289 (14.7)	38,619 (14.2)
3	95,851 (1.8)	10,867 (5.1)	14,323 (4.1)	22,834 (7.4)	20,058 (7.4)
4+	141,767 (2.7)	26,372 (12.3)	24,107 (6.9)	40,653 (13.2)	38,892 (14.3)
**Medical conditions, Mean ± SD**	0.5 ± 1.1	1.3 ± 2.1	1.0 ± 1.6	1.4 ± 2.6	1.6 ± 2.1
**Comorbidity score**					
0	4,653,358 (87.5)	149,691 (70.0)	267,237 (76.9)	184,545 (60.0)	164,730 (60.7)
1	219,171 (4.1)	19,143 (9.0)	28,089 (9.0)	31,014 (10.1)	28,580 (10.5)
2–3	296,335 (5.6)	23,855 (11.2)	31,264 (9.0)	49,656 (16.1)	40,638 (15.0)
4–5	79,517 (1.5)	10,051 (4.7)	10,757 (3.1)	18,872 (6.1)	16,522 (6.1)
6+	68,449 (1.3)	11,180 (5.2)	10,023 (2.9)	23,609 (7.7)	20,888 (7.7)
**Social deprivation**					
1 (Least deprived)	1,000,193 (18.8)	34,502 (16.1)	57,188 (16.5)	51,373 (16.7)	43,840 (16.2)
2	1,024,222 (19.3)	38,568 (18.0)	61,989 (17.9)	52,559 (17.1)	46,111 (17.0)
3	1,038,625 (19.5)	39,424 (18.4)	65,668 (18.9)	57,497 (18.7)	50,388 (18.6)
4	1,000,609 (18.8)	40,607 (19.0)	68,781 (19.8)	62,983 (20.5)	54,954 (20.3)
5 (Most deprived)	969,393 (18.2)	44,667 (20.9)	69,097 (19.9)	64,814 (21.1)	57,463 (21.2)
Missing	283,788 (5.3)	16,152 (7.6)	24,647 (7.1)	18,470 (6.0)	18,602 (6.9)
**Rurality**					
Non rural	4,207,825 (79.1)	150,372 (70.3)	274,378 (79.7)	258,919 (84.2)	222,419 (82.0)
Rural	1,074,234 (20.2)	62,647 (29.3)	69,256 (19.9)	47,766 (15.5)	47,963 (17.7)
Missing	34,771 (0.7)	901 (0.4)	1230 (0.4)	1011 (0.3)	976 (0.3)
**ED frequent visits**	213,920 (4.0)	213,920 (100.0)	38,684 (11.1)	51,047 (16.6)	70,530 (26.0)
**GP frequent visits**	348,238 (6.5)	38,684 (18.1)	347,370 (100.0)	51,345 (16.7)	119,344 (44.0)
**SP frequent visits** **Total frequent visits**	266,413 (5.8) 271,358 (5.1)	51,047 (23.9) 70,530 (33.0)	51,345 (14.8) 119,344 (34.4)	307,696 (100.0) 271,358 (88.2)	195,359 (72.0) 271,358 (100.0)

Abbreviation: *SD* Standard deviation; *ED* Emergency departments, *GP* General partitioners; *SP* Specialist physicians

^a^ Non frequent users are defined as 0–2 visits per year for ED, 0–6 visits per year for GP, 0–9 visits per year for SP and 0–16 visits per year for total ambulatory care services. Characteristics of non-frequent users can be obtained by subtracting the data of frequent users from the total population

^b^ The percentage corresponds to the total of the subgroup of frequent users. Frequent ED, GP, SP and total ambulatory care users represent respectively 4.0%, 6.5%, 5.8% and 5.1% of the total population

**Table 2:** Frequency of annual ambulatory care visits of the total population in Quebec, Canada for 2014–2015 (*n* = 5,316,830)

Number of visits	Emergency departments			General practitioners			Specialist physicians			Total ambulatory		
	Prevalence in general population	Number of visits	% of all visits	Prevalence in general population	Number of visits	% of all visits	Prevalence in general population	Number of visits	% of all visits	Prevalence in general population	Number of visits	% of all visits
Total	100.0	2,204,450	100.0	100.0	11,781,829	100.0	100.0	13,725,842	100.0	100.0	27,712,121	100.0
0	76.6	0	0.0	28.3	0	0.0	43.0	0	0.0	15.8	0	0
1	14.4	766,810	34.8	22.9	1,216,639	10.3	16.2	865,991	6.3	14.0	745,871	2.7
2	5.0	527,506	23.9	16.8	1,786,668	15.2	10.7	1,135,866	8.3	12.3	1,302,652	4.7
3–6	**3.6**	**715,805**	**32.5**	25.5	5,374,522	45.6	19.3	4,197,906	30.6	30.9	6,971,824	25.2
7–9	**0.1**	**113,227**	**5.1**	**4.0**	**1,661,263**	**14.1**	5.0	2,094,487	15.2	11.1	4,665,355	16.8
10–13	**0.3**	**47,312**	**2.1**	**1.8**	**1,037,985**	**8.8**	**2.9**	**1,750,128**	**12.8**	7.7	4,611,015	16.6
14–16	**0.0**	**12,949**	**0.6**	**0.4**	**343,356**	**2.9**	**1.1**	**843,888**	**6.1**	3.1	2,446,768	8.8
≥ 17	**0.0**	**20,841**	**1.0**	**0.3**	**361,396**	**3.1**	**1.8**	**2,837,576**	**20.7**	**5.1**	**6,968,636**	**25.2**

NOTE: Bold text for each ambulatory care service corresponds to the definition of frequent user. Frequent ED users represented 4.0% of the total population and consumed 41.3% of the total annual number of visits. Frequent GP users represented 6.5% of the total population and consumed 28.9% of the total annual number of visits. Frequent SP users represented 5.8% of the total population and consumed 39.6% of the total annual number of visits. Frequent users of total ambulatory care services represented 5.1% of the total population and consumed 25.2% of total ambulatory care services. Among the 6,968,636 visits made by frequent users of total ambulatory care services, 7.2% of visits were to an ED, 27.1% were to a GP and 65.7% were to a SP.

**Fig. 2:**
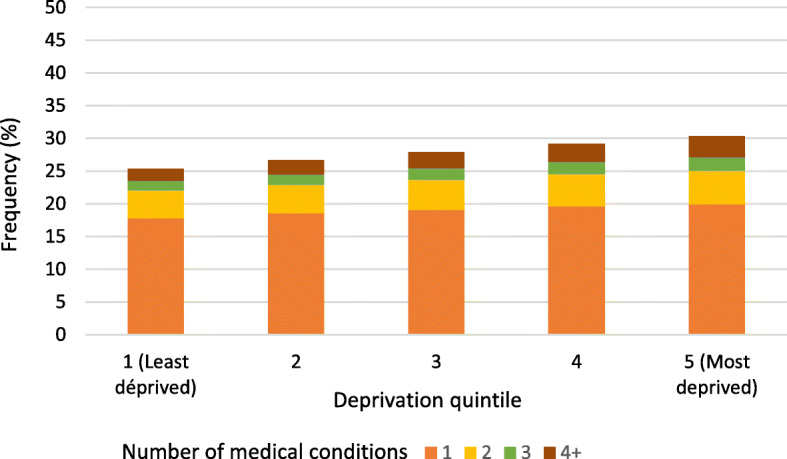
Frequency of medical conditions by deprivation quintile in the total population (*n* = 5,033,042). We excluded 283,788 inhatodividuals due to missing values for the deprivation index

In the entire population, the number of medical conditions increased constantly from the least deprived to the most deprived neighbourhoods (Fig. 2). While 74.6% of the least deprived group had no medical condition and 7.6% had at least 2, those proportions went to 69.7 and 10.4% respectively for the most deprived neighbourhoods.

**Fig. 3:**
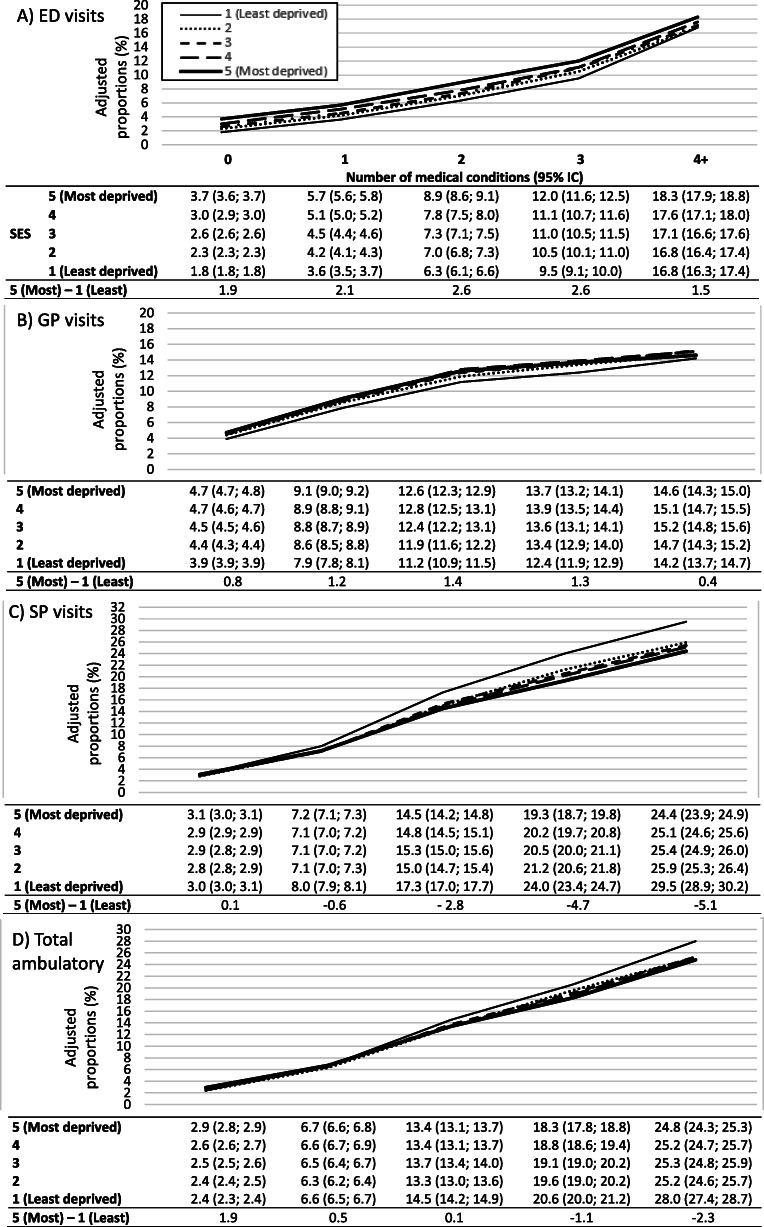
Adjusted proportions of frequent ambulatory care use [**a**) ED visits, **b**) GP visits, **c**) SP visits, **d**) Total ambulatory care services] by number of conditions, stratified by material deprivation quintile in the total population of Quebec (*n* = 5,033,042)

The potential effect modification of SES on the association between multimorbidity and frequent use of ambulatory care services is illustrated in Fig. 3. Within each category of ambulatory service, the adjusted proportion of frequent users increased with the number of medical conditions. For ED visits, the adjusted proportion of frequent users increased from 1.8 to 3.7% for the individuals without any medical conditions in the least and the most deprived groups, respectively, while in those with 4 or more medical conditions, it increased from 16.8 to 18.3% for individuals in the least and most deprived groups, respectively. The proportion of frequent ED users was higher among the most deprived but the absolute difference was approximately stable, ranging from + 1.9% to + 2.6% across the different categories of multimorbidity. However, a small decrease is worth noting at the highest level of multimorbidity (≥4 conditions) where the difference reaches its lowest level with + 1.5%. The proportion of frequent GP users increased rapidly up to two medical conditions, but much more slowly thereafter. The proportions of frequent GP visits were less important in the least deprived groups. Similar to ED visits, the absolute difference in frequent GP use between the individuals in first quintile of deprivation compared to the fifth quintile was stable up to ≥3 medical conditions, ranging from + 0.8% to + 1.4%, but reduced to + 0.4% in individuals with a disease count of 4 and more. The proportion of SP frequent users increased monotonically as the number of medical conditions increased, but contrary to ED and GP visits, the higher consumption of specialized services was observed in the least deprived group and the absolute difference increased with the number of medical conditions. Almost inexistent in individuals with no disease, the absolute difference between the most and least deprived reached − 5.1% in those affected by 4 or more diseases. This result suggests an effect modification by SES as the difference between the least deprived and the most deprived groups intensifies with the number of medical conditions. For total frequent ambulatory care utilization, we observed results similar to SP visits as the least deprived group has an overall higher use of services than the others.

The sensitivity analysis using the definition of frequent healthcare users set as the 80th percentile of all users showed similar results as the main analysis (not shown). The sensitivity analysis using a different multimorbidity measure yielded consistent results in terms of relationship between proportions of frequent users and the comorbidity score as well as in terms of effects of SES (See additional file 3). Only minor differences in the magnitude of the effects were observed mostly for GP and SP visits. For each ambulatory care service, the proportions of frequent users increased as the number of medical conditions increased and graphical positions of SES groups appeared in similar patterns. However, for the total frequent ambulatory care use, the widening gap between the least deprived and the most deprived group disappeared. Thus, the number of visits to the SP had a smaller impact on the total ambulatory care visits when multimorbidity was measured by the comorbidity score.

## Discussion

Our population-based cohort study demonstrates that the probability of being a frequent user of ambulatory care services increases with the increasing number of health conditions and is influenced by SES. Effect modification by SES is present for frequent SP users and is also reflected in frequent total ambulatory care use, as the gap between the most deprived group and the least deprived group widens with the number of health conditions. In our study, frequent users of ambulatory care (≈5%) used a disproportionate amount of care (≈25%), figures that correspond to previous research [11, 17]. Since only a small proportion of individuals were frequent users of all three categories of services, this may be an indicator that patterns of consumption of healthcare services vary between groups of frequent users. We also observed that SES seemed to have a smaller effect on frequent SP and total ambulatory care use for serious diseases (comorbidity score of 6+) as the difference in frequent SP use between the most and the least deprived groups was smaller than with the number of diseases. This smaller effect might be explained by the fact that serious diseases which have a higher comorbidity score are mostly diagnosed and managed by a SP. While a high number of conditions can represent the accumulation of less serious conditions for which the consultation of a SP depends on factors other than the conditions.

### Comparison with other studies

Our results concord with previous lines of evidence on multimorbidity and SES effect on ambulatory care use. The most socioeconomically deprived individuals tend to have more chronic conditions than the least deprived ones [7, 8, 32]. Even though any specific causes are yet to be identified, a higher concentration of risk factors such as lifestyle factors (smoking, poor alimentation or lack of exercise) or a poorer healthcare are possible hypotheses [4]. In addition to being a consequence of aging, multimorbidity is also believed to be a consequence of environmental risks factors associated with SES and social marginalization such as dependency, ethnic-concentrated communities or residential instability [33]. While it is well known that a low SES is associated with a greater use of ED and GP whereas a high SES is associated with a greater use of SP [11, 21], our findings demonstrate that the least deprived individuals are also more likely to be frequent users of ambulatory care services. Similar to other studies, we showed that the proportion of visits to each category of services increased with the number of medical conditions [19, 34–36]. A Canadian study showed that multimorbidity was associated with almost twice the odds of visiting the ED in comparison to individuals without multimorbidity (OR = 1.8, 95% CI = 1.4–2.2) [37]. An increase in the number of ED visits may reflect the ineffectiveness of care in managing the complicated needs of multiple chronic diseases or acute exacerbations [38, 39]. Additionally, the number of visits to a GP increases with the number of diseases since patients with multimorbidity often require a closer medical follow-up than those with only one or no chronic disease [35]. Also, it is more likely for a patient with multimorbidity to consult a specialist physician for a disease that otherwise could have been treated by primary care services [6].

In a universal healthcare system that is intended to remove barriers to access to health services and to aim at reducing inequalities in health, [40] SES still seems to play a role in the utilization of healthcare services for individuals with multimorbidity. Individuals with a lower SES seemed to be more frequent users of ED which might be an indicator of a potential gap in continuity of care [41, 42]. Those results may be explained by several factors including a lack of knowledge on healthcare systems, a perceived lack of rapid access to quality care or a lack of access to alternative primary care services [41, 43]. The fact that disparities in frequent ED visits between the different socioeconomic groups did not amplify with the number of chronic diseases is reassuring as individuals seemed to be receiving proportional care according to their health status. SES and the presence of chronic diseases seemed to have independent effect on ED and GP use as we did not observe a significant interaction between the two. Absence of effect modification of SES in the association between the number of chronic conditions and GP visits is coherent with a study by Olah and al. (2013). The authors did not observe an interaction between the number of chronic conditions and SES regarding the likelihood of a patient to receive a positive response when seeking an appointment with a GP as those two factors had independent effect [44].

Effect modification of SES in the association between the number of medical diseases and frequent SP visits indicated a disparity between the least deprived group and the most deprived groups as the number of chronic diseases increased. In Quebec, only the first referral to a SP is made by a GP. SP services are mostly as accessible as GP services except for rural communities where the distance to specialized care might represents a barrier to this care. The fact that the least deprived group consulted more SP without consulting more GP shows that there is a preferential access to this service for the individuals with higher SES. A study conducted in Scotland showed that multimorbid patients coming from less deprived areas received longer consultations than non-multimorbid patients coming from the same area, while multimorbid patients coming from more deprived areas had a similar consultation length to non-multimorbid patients [45]. GPs in richer areas were also perceived to be more emphatic and attentive towards individuals with higher SES [45]. Furthermore, studies have shown that a patient’s attitude and expectation can have an impact on their referral to specialists [21, 46]. Individuals who are less educated and poorer may have difficulties communicating their healthcare needs [21]. This health literacy problem is associated with a lower SES, belonging to an ethnic community or living in rural areas [47] and represents a barrier to accessing specialized care. Besides, low health literacy mediates the relationship between SES and health behaviors among others [47].

Finally, individuals with higher SES group may be more critical towards an intervention in primary care and perceived more easily the benefit that would come from a SP visit [12]. There is a need to explore whether this phenomenon could potentially lead to overdiagnosis, overtreatment or overtesting [48] as the higher cut-off (≥10 annual visits) for frequent SP users can highlight a potential problem of overconsumption of specialized care. It is worth noting that 5% of the population made 10 or more visits to the SP, while only 2.5% and 0.3% of the population made the same number of visits to a GP or an ED respectively which can demonstrate that the system is oriented towards a non-integrative medicine where care is more disease-oriented than patient-oriented [49]. Perhaps, some visits that could have been made with a GP are transferred to a SP especially when a patient suffers from multiple chronic conditions. These aspects should be further explored especially from a cost-perspective for the health system.

### Strengths and limitations

To our knowledge, this is the first population-based cohort study to examine the effect modification of SES in the association between multimorbidity and frequent ambulatory care use. This study included a large number of diseases and is one of the few to explore the impact of multimorbidity using two different methods to define the concept. Also, the three outcomes allowed to study the use of healthcare on a continuum of health services going from primary to specialized care. This study has limitations some of which are due to the databases we used [22]. First, we only included a limited number of covariates based on their availability in our databases. We excluded certain groups such as individuals living in long-term care facilities and native nations due to a lack of information. Even if those individuals represent a relatively small proportion of the Quebec population, they often have characteristics that are distinct from the rest of the population. Those groups often have more chronic diseases and particular socioeconomic characteristics. Secondly, we could not assess the impact of healthcare professionals who are not included in the fee-for-service billing system (e.g. psychologist, physiotherapist, social workers, etc.). Thirdly, it was not possible to account for disease severity which may differ across socioeconomic gradients and may have impacted health seeking behaviors. Finally, we used a deprivation index as an estimate of the SES for small areas and not an individual measure. Therefore, it can have underestimated the extent of social inequalities, especially for larger areas. However, this deprivation index is a validated measure widely used in the public health field in the province [26].

## Conclusions

This population-based study demonstrates that SES modifies the association between multimorbidity and frequent use of SP in Quebec. Our conclusions could be inferred to populations with comparable characteristics and healthcare system. Individuals with a lower SES visit more primary care services such as ED and GP but globally the difference in utilization between the most deprived and the least deprived does not increase as the number of chronic conditions increases. However, despite the universal health care system, individuals with a lower SES are less likely to visit a SP even when visiting primary care services more often. In fact, SES seems to contribute to a differential access to SP services for the least deprived individuals. Further studies are needed to better understand the underlying causes of this differential access to specialist care and to reduce health inequalities.

## Supplementary Information

**Additional file 1:.** Flow chart of study inclusion and exclusion criteria. This additional file contains the Fig. 1 of the manuscript.

**Additional file 2: **Codes used to identify healthcare providers and ambulatory care **services.** This additional file contains the description of the method used to identify healthcare providers and ambulatory care services in our study.

**Additional file 3: **Adjusted proportions of frequent ambulatory care use [A) ED visits, B) GP visits, C) SP visits, D) Total ambulatory care services] by comorbidity score, stratified by material deprivation quintile in the total population of Quebec (*n* = 5,033,042). This additional file contains the result of the sensitivity analyses.

## Data Availability

The datasets generated and/or analyzed during the current study are not publicly available due to data confidentiality requirements from the QICDSS but are available from the corresponding author on reasonable request.

## Abbreviations

CI: Confidence interval
ED: Emergency departments
GP: General practitioners
OR: Odds ratio
QICDSS: Quebec integrated chronic disease surveillance system
SD: Standard deviation
SAS: Statistical analysis system
SES: Socioeconomic status
SP: Specialist physicians
