# Supplementary material for: Does socio-economic status influence the effect of multimorbidity on the frequent use of ambulatory care services in a universal healthcare system? A population-based cohort study
Source: BMC Health Serv Res. 2021 Mar 6;21:202. doi: 10.1186/s12913-021-06194-w (PMC7937264; doi:10.1186/s12913-021-06194-w)
Supplement: Supplementary file 1 — Additional file 1:. Flow chart of study inclusion and exclusion criteria. This additional file contains the Fig. 1 of the manuscript. [file 12913_2021_6194_MOESM1_ESM.docx]

Number of individuals in the database between fiscal year 2012 and 2016

N = 5,486,157

**Excluded Included**

N = 5,427,576

N = 58,581

Aged < 18 years on Oct 1st 2014

N = 5,344,870

N = 82,706

Individuals deceased between April 1^st^

2015 and March 31^st^ 2016

N = 5,316,830

N = 28,040

Individuals living in long-term health

care facilities

Final cohort (Total population)

N = 5,516,830

**Additional file 1: Flow chart of study inclusion and exclusion criteria**. A fiscal year begins on April 1^st^ and ends on March 31^st^.
