# Supplementary material for: Does socio-economic status influence the effect of multimorbidity on the frequent use of ambulatory care services in a universal healthcare system? A population-based cohort study
Source: BMC Health Serv Res. 2021 Mar 6;21:202. doi: 10.1186/s12913-021-06194-w (PMC7937264; doi:10.1186/s12913-021-06194-w)
Supplement: Supplementary file 2 — Additional file 2: Codes used to identify healthcare providers and ambulatory care services. This additional file contains the description of the method used to identify healthcare providers and ambulatory care services in our study. [file 12913_2021_6194_MOESM2_ESM.docx]

Additional file 2: Codes used to identify healthcare providers and ambulatory care services

| **Healthcare services** | **Codes** |
| --- | --- |
| Emergency room department | Institution code = 0X7 |
| General practitioners | Provider code = 1  Institution code = 000, 0X1, 0X7, 512, 52X, 53X, 54X, 55X, 56X, 8X5, 9X2 |
| Specialist physicians | Provider code = 2  Institution code = 000, 0X1, 0X7, 512, 52X, 53X, 54X, 55X, 56X, 8X5, 9X2 |

Consultations with a general practitioner or a specialist physician were determined using the provider code variable (1 = general practitioner, 2 = specialist doctor) and the code of institution. A general practitioner is defined as a physician registered to the Quebec Federation of General Practitioners (FMOQ) and a specialist physician is one registered to the Quebec Federation of Medical Specialist (FMSQ). Only the medical services billed in private practice, community health centers or outpatient clinics were considered.
