# Supplementary material for: Does socio-economic status influence the effect of multimorbidity on the frequent use of ambulatory care services in a universal healthcare system? A population-based cohort study
Source: BMC Health Serv Res. 2021 Mar 6;21:202. doi: 10.1186/s12913-021-06194-w (PMC7937264; doi:10.1186/s12913-021-06194-w)
Supplement: Supplementary file 3 — Additional file 3: Adjusted proportions of frequent ambulatory care use [A) ED visits, B) GP visits, C) SP visits, D) Total ambulatory care services] by comorbidity score, stratified by material deprivation quintile in the total population of Quebec (n = 5,033,042). This additional file contains the result of the sensitivity analyses. [file 12913_2021_6194_MOESM3_ESM.docx]

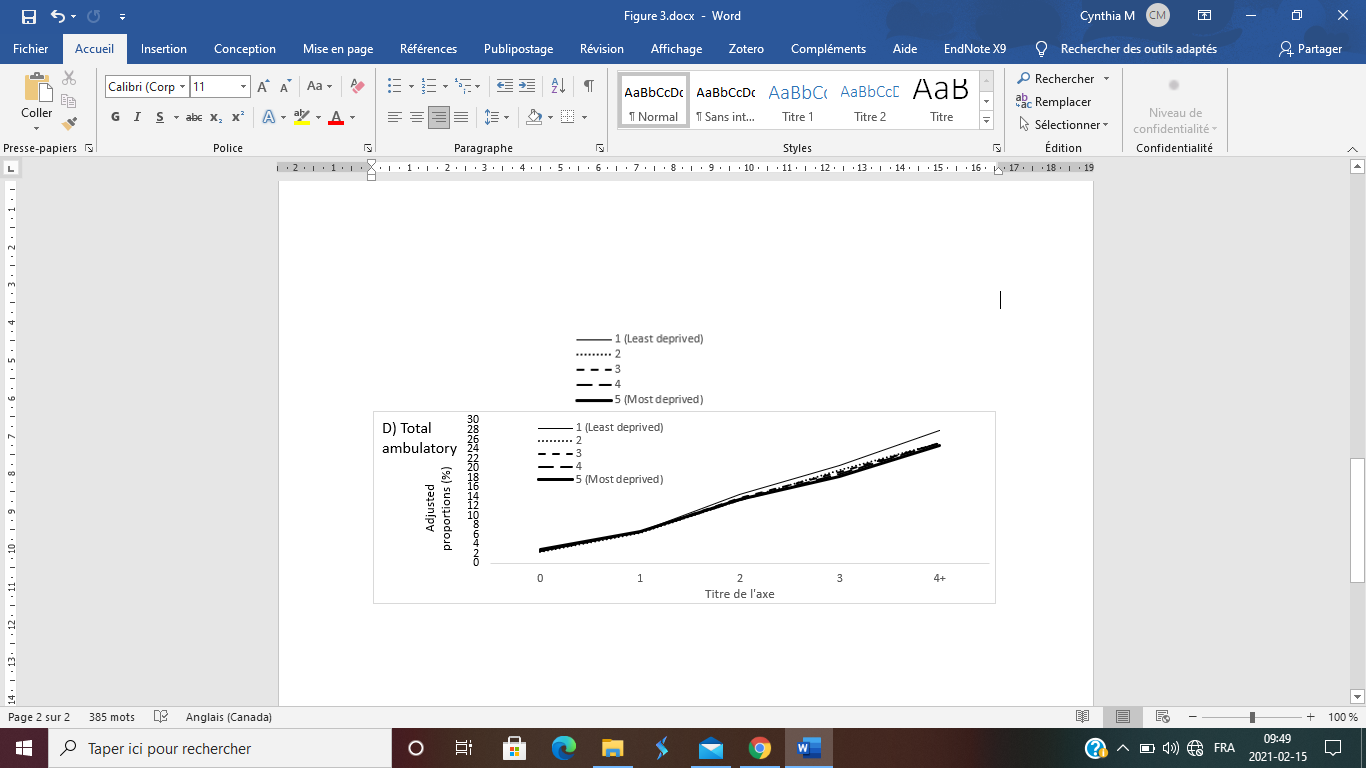


|  | | **0** | **1** | **2-3** | **4-5** | **6+** |
| --- | --- | --- | --- | --- | --- | --- |
|  | | **Comorbidity score (95% IC)** | | | | |
| **SES** | **5 (Most deprived)** | 4.1 (4.1; 4.2) | 9.6 (9.3; 9.8) | 8.8 (8.5; 9.0) | 12.7 (12.2; 13.2) | 15.7 (15.1; 16.3) |
|  | **4** | 3.4 (3.4; 3.4) | 8.6 (8.3; 8.8) | 7.7 (7.5; 8.0) | 11.4 (10.9; 11.9) | 14.3 (13.8; 14.9) |
|  | **3** | 3.0 (2.9; 3.0) | 7.8 (7.6; 8.1) | 7.1 (6.9; 7.3) | 10.4 (9.9; 10.9) | 14.4 (13.8; 14.9) |
|  | **2** | 2.6 (2.6; 2.7) | 7.2 (7.0; 7.5) | 6.8 (6.6; 7.0) | 10.5 (10.0; 11.0) | 13.3 (12.7; 13.9) |
|  | **1 (Least deprived)** | 2.1 (2.1; 2.1) | 6.0 (5.8; 6.3) | 5.8 (5.6; 6.0) | 9.6 (9.1; 10.1) | 13.2 (12.6; 13.9) |
| **5 (Most) – 1 (Least)** | | 2.0 | 3.6 | 3.0 | 3.1 | 2.5 |

| **5 (Most deprived)** | 5.8 (5.7; 5.8) | 11.7 (11.4; 12.0) | 9.1 (8.9; 9.4) | 11.2 (10.7; 11.6) | 12.4 (11.9; 12.4) |
| --- | --- | --- | --- | --- | --- |
| **4** | 5.7 (5.6; 5.7) | 11.3 (11.0; 11.5) | 9.2 (8.9; 9.4) | 11.2 (10.7; 11.7) | 11.9 (11.4; 12.4) |
| **3** | 5.5 (5.5; 5.6) | 11.3 (11.0; 11.6) | 8.8 (8.6; 9.0) | 10.7 (10.3; 11.2) | 11.8 (11.2; 12.3) |
| **2** | 5.3 (5.3; 5.4) | 10.5 (10.3; 10.8) | 8.5 (8.3; 8.7) | 10.4 (10.0; 10.9) | 11.0 (10.5; 11.5) |
| **1 (Least deprived)** | 4.7 (4.7; 4.8) | 9.8 (9.5; 10.1) | 7.6 (7.4; 7.8) | 9.6 (9.2; 10.1) | 10.1 (9.6; 10.7) |
| **5 (Most) – 1 (Least)** | 1.1 | 1.9 | 1.6 | 1.6 | 2.3 |

| **5 (Most deprived)** | 3.9 (3.9; 4.0) | 11.7 (11.4; 12.0) | 13.4 (13.2; 13.7) | 18.8 (18.3; 19.4) | 27.7 (26.9; 28.4) |
| --- | --- | --- | --- | --- | --- |
| **4** | 3.7 (3.7; 3.8) | 11.3 (11.1; 11.6) | 13.1 (12.9; 13.4) | 18.9 (18.3; 19.5) | 28.3 (27.6; 29.1) |
| **3** | 3.7 (3.6; 3.7) | 11.5 (11.3; 11.8) | 13.4 (13.1; 13.7) | 18.5 (17.9; 19.1) | 28.4 (27.6; 29.2) |
| **2** | 3.6 (3.5; 3.6) | 11.2 (10.9; 11.5) | 13.2 (13.0; 13.5) | 18.6 (18.0; 19.3) | 29.0 (28.2; 29.8) |
| **1 (Least deprived)** | 3.8 (3.7; 3.8) | 12.6 (12.3; 12.9) | 14.5 (14.2; 14.8) | 20.6 (19.9; 21.2) | 31.4 (30.5; 32.2) |
| **5 (Most) – 1 (Least)** | 0.1 | -0.9 | - 0.9 | -1.8 | -3.7 |

| **5 (Most deprived)** | 3.7 (3.7; 3.8) | 11.6 (11.3; 11.9) | 11.7 (11.5; 12.0) | 17.4 (16.8; 18.0) | 26.1 (25.4; 26.9) |
| --- | --- | --- | --- | --- | --- |
| **4** | 3.5 (3.4; 3.5) | 11.1 (11.8; 11.3) | 11.5 (11.2; 11.7) | 17.1 (16.5; 17.7) | 25.6 (24.9; 26.4) |
| **3** | 3.3 (3.3; 3.4) | 11.0 (10.7; 11.3) | 11.3 (11.0; 11.5) | 16.8 (16.3; 17.4) | 25.8 (25.1; 26.6) |
| **2** | 3.2 (3.1; 3.2) | 10.5 (10.2; 10.8) | 11.1 (10.9; 11.4) | 16.7 (16.1; 17.3) | 25.1 (24.4; 25.9) |
| **1 (Least deprived)** | 3.1 (3.1; 3.1) | 10.9 (10.6; 11.2) | 11.3 (11.1; 11.6) | 17.3 (16.7; 17.9) | 26.9 (26.1; 27.7) |
| **5 (Most) – 1 (Least)** | 0.6 | 0.7 | 0.4 | 0.1 | -0.8 |

Additional file 3: Adjusted proportions of frequent ambulatory care use [A) ED visits, B) GP visits, C) SP visits, D) Total ambulatory care services] by comorbidity score, stratified by material deprivation quintile in the total population of Quebec (n = 5,033,042).
